# Heart Team for Left Atrial Appendage Occlusion: A Patient-Tailored Approach

**DOI:** 10.3390/jcm11010176

**Published:** 2021-12-29

**Authors:** Stefano Branzoli, Fabrizio Guarracini, Massimiliano Marini, Giovanni D’Onghia, Daniele Penzo, Silvio Piffer, Dimitri Peterlana, Angelo Graffigna, Michele Massimo Gulizia, Sandro Gelsomino, Mark La Meir

**Affiliations:** 1 Department of Cardiac Surgery, UZ Brussel, Av. du Laerbeek 101, 1090 Brussels, Belgium; sandro.gelsomino@maastrichtuniversity.nl (S.G.); mark.lameir@uzbrussel.be (M.L.M.); 2 Cardiac Surgery Unit, Santa Chiara Hospital, Largo Medaglie d’oro, 38122 Trento, Italy; angelo.graffigna@apss.tn.it; 3 Department of Cardiology, Santa Chiara Hospital, Largo Medaglie d’oro, 38122 Trento, Italy; fabrizio.guarracini@apss.tn.it (F.G.); massimiliano.marini@apss.tn.it (M.M.); giovanni.donghia@apss.tn.it (G.D.); 4 Department of Anesthesia, Santa Chiara Hospital, Largo Medaglie d’oro, 38122 Trento, Italy; daniele.penzo@apss.tn.it; 5 Neurology Unit, Santa Chiara Hospital, Largo Medaglie d’oro, 38122 Trento, Italy; silvio.piffer@apss.tn.it; 6 Division of General Internal Medicine Santa Chiara Hospital, Largo Medaglie d’oro, 38122 Trento, Italy; dimitri.peterlana@apss.tn.it; 7 Cardiology Complex Unit, Garibaldi Nesima Hospital, 95122 Catania, Italy; michele.gulizia60@gmail.com; 8 Heart Care Foundation, 50121 Florence, Italy

**Keywords:** heart team, left atrial appendage occlusion, contraindication oral anticoagulants, percutaneous procedure, thoracoscopic surgery

## Abstract

Background and Purpose: Left atrial appendage occlusion (LAAO) is an accepted therapeutic option for stroke prevention; however, the ideal technique and device have not yet been identified. In this study we evaluate the potential role of a heart team approach for patients contraindicated for oral anticoagulants and indicated for left atrial appendage closure, to minimize risk and optimize benefit in a patient-centered decision-making process. Methods: Forty patients were evaluated by the heart team for appendage occlusion. Variables considered were CHA_2_DS_2_VASc, HASBLED, documented blood transfusions, comorbidities, event forcing anticoagulant interruption, past medical history, anatomy of the left atrial appendage, and patient quality of life. Twenty patients had their appendage occluded percutaneously (65% male, mean age 72.3 ± 7.5, mean CHA_2_DS_2_VASc 4.2 ± 1.5, mean HASBLED 3.5 ± 1.1). The other twenty underwent thoracoscopic occlusion (65% male, mean age of 74.9 ± 8, mean CHA_2_DS_2_VASc 6.0 ± 1.5, HASBLED mean 5.4 ± 1.4). Percutaneous patients were on dual antiplatelet therapy for the first three months and aspirin thereafter, whereas the others received no anticoagulant/antiplatelet therapy from the day of surgery. Follow up included TEE, CT scan, and periodical clinical evaluation. Results: Mean duration of procedures and hospital stay were comparable. All patients had complete exclusion of the appendage; at a mean follow up of 33.1 ± 14.1 months, no neurological or hemorrhagic events were reported. Conclusions: A heart team approach may improve the decision-making process for stroke and hemorrhage prevention, where LAAO is a therapeutic option. Percutaneous and thoracoscopic appendage occlusion seem to be comparably safe and effective. An epicardial LAAO could be advisable in patients for whom the risk of bleeding is estimated as being too high for post-procedural antiplatelet therapy.

## 1. Introduction

The left atrial appendage (LAA) is known for being the principal site of thrombi formation in patients with atrial fibrillation (AF) [1]. The ESC, in collaboration with EACTS 2020 guidelines for atrial fibrillation, recommend oral anticoagulation (OAC) with a CHA_2_DS_2_VASc >2 (class I indication); if contraindications to long term OAC are present, left atrial appendage occlusion (LAAO) might be considered (class IIb recommendation) [2].

The majority of patients undergoing surgery for atrial fibrillation will have their LAA addressed. Excision with suture closure has been part of the Maze operation since the first report in 1987 [3]. More recently, alternative closure techniques have been introduced using endocardial or epicardial over-sewing, excision, ligation, stapling, or application of a clip system (AtriClip, AtriCure Inc., Mason, OH, USA). Although part of the surgical routine in most centers, several studies have raised concerns about the outcomes of these surgical techniques [4,5,6,7]. For standalone, totally thoracoscopic procedures, LAA occlusion (LAAO-T) clipping seems to be the most promising closure device, in terms of safety and efficacy [8,9]. In the multicenter cohort analysis of Van Laar et al. [8], successful LAA closure was reported in 95.0% of cases, with no intraoperative or clip-related complications, and an overall 30-day freedom from any complication rate of 96.4%.

Several percutaneous devices are available for occlusion of the LAA (LAAO-P), the most frequently implanted and studied is the Watchman (Boston Scientific Corp, Minneapolis, MN). Protect AF, Prevail, Ewolution trials, and others [10] have documented excellent closure rates (98.5%), along with the presence of factors leading to ineligibility for, or predictive of, suboptimal device implantation in up to 10% of patients. Presence of residual leaks up to 8% at six months; periprocedural complications of 2.8–8.7%; bleeding of 2.6%; need of anticoagulant therapy at 1–2 years in 6.8% and 8% of patients, respectively; and need of dual antiplatelet therapy (DAPT) in 7% of patients at 2 years have raised questions about technical aspects, the clinical impact of peri-device leaks, and post procedure anticoagulant therapy [11,12]. The documentation of device thrombosis, up to 4.1%, and an increased rate of bleeding complications in patients with HASBLED > 3 [13] have raised concerns on the ideal timing to stop mandatory post procedural anticoagulant/antiplatelet therapy, especially in the presence of comorbidities with a high risk of blood transfusion [13,14,15]. Predicting clinical and bleeding outcomes in frail patients is difficult and even more challenging in those with contraindications or poor tolerance to the anticoagulants eligible for LAAO; for this reason a heart team approach has been advocated [16], and there seems to be growing interest in this topic, as recently reported by Kany et al. [17]. Since LAAO-T with a clip does not introduce any foreign materials inside the left atrium (chamber with low flow blood velocity), there should be no need for post-operative antiplatelet therapy (APT). This might address some of the post-procedural bleeding issues, could play a major role in improving patients’ health outcomes, and guide patient selection for both treatment strategies. 

Here, we present our experience and the clinical impacts of a heart team approach to patient selection for LAA exclusion, either with a standalone, totally thoracoscopic, or a percutaneous procedure. This was carried out to minimize the risk and optimize the outcome for each individual patient.

## 2. Materials and Methods

Forty patients with AF and a contraindication to oral anticoagulants underwent heart team evaluation for left atrial appendage exclusion for systemic embolism and hemorrhage prevention between 2017 and 2020 (Table 1).

The team met on a monthly basis and was composed of two electrophysiologists, a cardiac surgeon, an anesthesiologist, a neurologist, and a referring physician. All of the participating patients had a history of bleeding, requiring treatment/intervention and/or cardioembolic event, CHA_2_DS_2_VASc > 2, HASBLED > 1. Patients were contraindicated anticoagulation for the following reasons: cerebral hemorrhage (*n* = 12), non-cerebral life threatening hemorrhage (*n* = 12), non-life-threatening repetitive bleeding (*n* = 10), or having an underlying condition associated with high bleeding risk (2 Rendu–Osler–Weber syndrome, 2 cerebral amyloid angiopathy, 2 myelodysplastic syndrome). Criteria for exclusion from the study included previous PTCA (<1 year), comorbidity with life expectancy <1 year, heart disease requiring combined procedure, symptomatic carotid disease, and presence of left atrial appendage thrombus.

**Figure 1:**
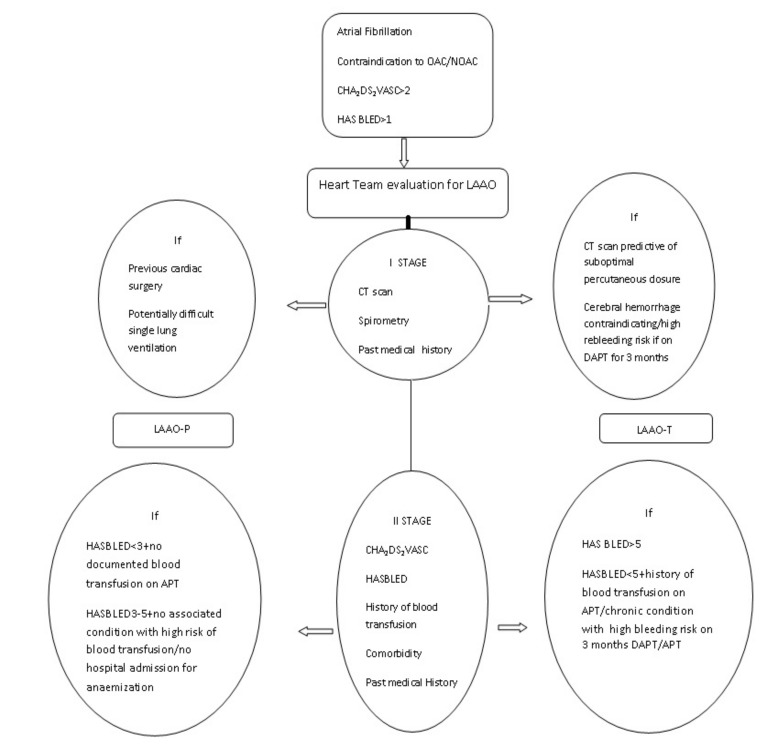
Flow chart. Two stages decision making process.

The variables identified for directing patients to a surgical or percutaneous group were past medical history, underlying condition that contraindicated oral anticoagulation and necessitated its interruption, risk of bleeding recurrence, history of blood transfusion, anatomy of the appendage on CT scan, lung function, nature of the acute/recent hemorrhagic event, expected time for antiplatelet/anticoagulation reintroduction, CHA_2_DS_2_VASc, HASBLED [18], and patient quality of life expectancy. All these variables were arbitrary identified by all members of the team, prespecified and selected as considered directly affecting procedural and clinical outcomes based on common clinical practice, the expertise of each member of the team [17], and published reports on LAAO [7,8,9,10,11,12,13,14]. In the first stage of evaluation, the variables considered were CT scan report, lung function, and past medical history, as considered strictly predictive of procedure feasibility and success of device implantation; all other criteria were considered in the second stage for each patient for either procedure, in an attempt at optimizing further the procedural success rate, decreasing peri-procedural complications, and reducing hospitalization and anaemization requiring further treatment/hospitalization at follow up. In case of HASBLED >5 a LAAO-T procedure was preferred, since DAPT was considered to have a negative prognostic effect, due to the lack of an estimated bleeding risk (maximum 12.5 bleeds per 100 patients/year with HASBLED = 5) [18] (Figure 1).

Preoperative investigations included a CT scan (Siemens Somatom Definition AS) for procedural aspects and appendage classification, in accordance with Di Biase et al. [19] (Table 2), transthoracic and transesophageal echocardiography (Philips iE33), to rule out intracavitary thrombi and concomitant heart disease requiring combined procedure, a baseline bilateral carotid doppler ultrasound for neurological follow up, and spirometry to detect potential single/double lung ventilation issues required for thoracoscopic and percutaneous procedures respectively. If suspicion of significant coronaropathy was raised at CT scan, an EKG exercise test or stress echocardiography was planned.

To allow the heart team members to be involved in both treatment options, percutaneous and thoracoscopic procedures were scheduled on the same day. Both procedures were performed under general anesthesia, and the same anesthesiology protocol (with bilateral lung ventilation in the LAAO-P group and right single lung ventilation in the LAAO-T group) was followed. All device deployments were guided by transesophageal echo and additional fluoroscopy or direct-view in percutaneous or thoracoscopic approach respectively. Approval was sought from the appointed hospital’s ethics committee (A391-11-2020), and informed consent was obtained from the patients.

### 2.1. Statistics

Categorical variables are reported with numbers and percentages, continuous variables with mean ± SD, median and IQR (Q1–Q3). For statistical analysis the Shapiro–Wilk test was used to test the normality of the continuous variables, the Wilcoxon rank sum test was used to compare groups of nonparametric data. The comparisons between groups of categorical data were performed using a Chi square test if expected cases were at least five, or Fisher exact test if expected cases were less than five. The significance level was set at 0.05 for all analysis, and statistical analysis was performed using SAS version 9.4

### 2.2. LAAO-T Procedure

The procedure was performed as described previously [20]. Briefly, with the patient in supine position, standard monitoring system and defibrillation pads, three 12-mm ports in a ‘hockey stick’ figure were placed: in the fifth intercostal space along the mid axillary line, third intercostal space along the anterior axillary line, and sixth–seventh intercostal space between the anterior and mid axillary line. After CO_2_ insufflation and opening the pericardium, the LAA was measured with a dedicated sizing-tool and the AtriClipPro2 was positioned under direct view and TEE guidance.

### 2.3. LAAO-P Procedure

Under general anesthesia and standard monitoring, the Watchman device (Boston Scientific Corp, Marlborough, MA, USA) was implanted at the ostium of the LAA via a transseptal puncture and through femoral access, as described [21].

### 2.4. LAAO-T and LAAO-P Procedures: Role of Echo Imaging

Echocardiography plays an important role when LAAO is a therapeutic option, not only during the pre-procedural phase, to detect the presence of LAA thrombus or anatomical aspects influencing percutaneous device selection [22], but also during the intra-operative and post-operative phases. During device delivery, TEE is critical in both LAAO-T and LAAO-P, with the widely accepted views at mid esophageal level in 0°, 45°, 90°, and 135° planes [20,22]. In LAAO-P, real-time TEE is also helpful for guiding interatrial septum puncture, and additional 3D multi-planar reconstruction is useful for adequate evaluation of the landing zone, measurement of 8–20% device compression, and assessment of a tight seal under concomitant fluoroscopic views and contrast angiography [22]. In the follow up, the role of echocardiography is of paramount importance, to document the device stability and detection of device-related thrombosis and peri-device leakage, influencing subsequent therapeutic decisions [22]. Notably, in the rapidly evolving field of echo imaging applied to percutaneous procedures, there is a growing interest in intracardiac echocardiography (ICE), which, with a reproducible three-views approach, as reported by Patel et al., and not requiring general anesthesia, might contribute to simplifying the procedure, with equally satisfactory results [23].

### 2.5. Post Procedural Anticoagulation Regimen

In the LAAO-P group, dual antiplatelet therapy for three months and aspirin 100 mg/day thereafter were prescribed.

In the LAAO-T group heparin, anticoagulants and antiplatelet therapy were stopped on the day of the procedure and no further therapy was prescribed thereafter.

### 2.6. Follow Up

All patients attended an outpatient clinic visit, including full physical examination and completion of a QVSFS questionnaire (questionnaire for verifying stroke-free status (QVSFS)) [24] at 1, 3, 6, and 12 months, and annually thereafter.

**Figure 2:**
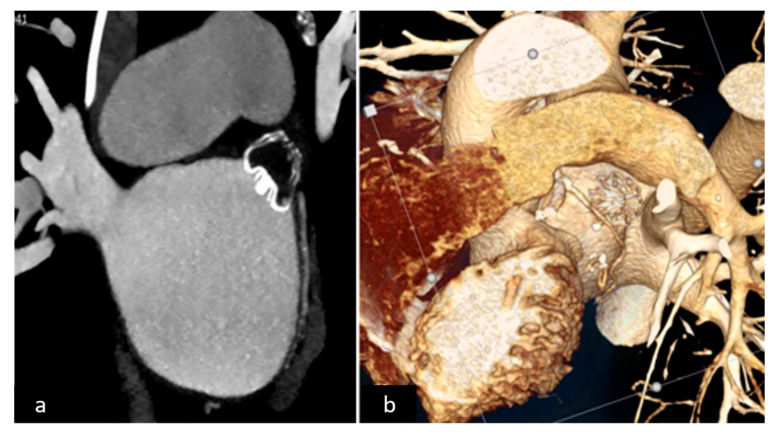
(**a**) 2D CT scan endocardial device, (**b**) 3D CT scan reconstruction.

**Figure 3:**
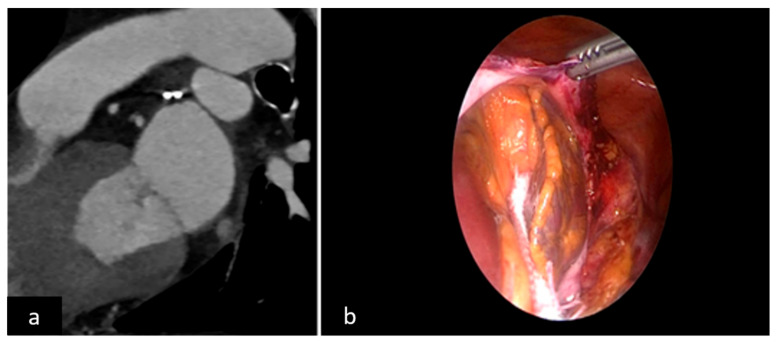
(**a**) 2D CT scan epicardial closure, (**b**) surgical view.

For appendage closure assessment, TEE at 1 month and CT scan at 3 months were planned for leak detection and stump measurement in both groups. Criteria of success in the watchman group were absence or presence of minor and major leaks of less or more than 5 mm, respectively, in accordance with Wunderlich et al. [10] (Figure 2a,b). In the TT group a successful result was considered a stump less than 1 cm, in accordance with Emmert et al. [25] (Figure 3a,b).

## 3. Results

All forty patients underwent appendage closure. One patient initially scheduled for percutaneous approach was transferred to surgery, due to an LAA anatomy unsuitable for a percutaneous device, which became evident during the procedure. At the end of the study, twenty patients were enrolled in each group. History of previous cerebral hemorrhage and comorbidity with high bleeding risk on APT (e.g., Rendu–Osler–Weber syndrome, diffuse GI angiodysplasia, cerebral amyloid angiopathy, or cerebral cavernomas) were more common in the LAAO T group, as HASBLED and CHA_2_DS_2_VASc were higher, and history of non-cerebral non-GI bleeding was more common in the LAAO-P group; whereas, previous GI bleeding and all other variables were equally distributed among the two groups (Table 1).

Mean duration of the procedure was 54.4 ± 12.7 min in the LAAO-P and 52.2 ± 14.3 min in the LAAO-T group. Post-procedure mean ventilation time was 11.2 ± 6.4 min for LAAO-P and 15.8 ± 16.4 for LAAO-T, with mean hospital stay of 3.4 ± 0.7 in LAAO-P and 3.8 ± 0.9 days in LAAO-T. No major complications occurred in either of the two treatment groups: with one pericarditis in the LAAO-T group, and one groin hematoma in the LAAO-P group (Table 3).

All patients were returned to the ward after the procedure, except for one patient in the LAAO-T group, who required slow respiratory weaning, due to COPD. No procedure related pulmonary morbidity or deaths were documented perioperatively or during follow up. One patient who was eligible for both procedures was treated surgically, due to her preference. The anatomies of the appendages were comparable between the two groups (Table 2). TEE and CT scans showed excellent device deployment in all cases, with a mean stump of 3.5 ± 3.0 in the LAAO-T group, and one case of minor leak (<5 mm) in the LAAO-P group. No device displacement was reported during follow up. Blood loss was negligible, no blood transfusions were needed, and no wound complications occurred. Before the procedure 27 patients had been admitted for anemia, with 17 receiving blood transfusions; of those, 13 were enrolled in LAOO-T, since antiplatelet therapy was deemed to have a negative prognosis in the long term.

Follow up was complete for all 40 patients (mean 33.1 ± 14.1). No hospital readmission was documented for cardiovascular or neurological events, and in QVSFS consultations no suspicions of neurological events were reported.

## 4. Discussion

The LAA is known to be an important source of thrombi formation, leading to strokes or transient ischemic attack. Anticoagulants are highly effective, but with anti-vit K there are concerns regarding interactions, the challenges associated in maintaining therapeutic levels, and patient compliance [26], and the prescription of NOACs is limited by restrictions and a discontinuation rate of 17–28%, as shown by the ARISTOTLE, ROCKET, and RE-LY trials [27,28]. Data available to date suggest that long term anticoagulation is not prescribed in up to 30% of patients worldwide [29], and in the case of life-threatening hemorrhage, there is still a debate about when to restart anticoagulant therapy [15]. Bleeding remains a risk with any antithrombotic therapy, with a large proportion of patients remaining undertreated, as show in the GLORIA–AF study [30]. Despite the promising added value of machine learning models to better predict and further improve outcomes in frail patients predisposed to hemorrhagic events, as recently reported by Sarajlic et al. [31] alternative therapeutic strategies continue to be researched.

The purpose of a heart team is to utilize multidisciplinary expertise in decision-making for the treatment of complex patients. To our knowledge, this is the first heart team guided approach for left appendage exclusion in patients contraindicated for OAC/NOAC, considering a percutaneous or thoracoscopic approach. We hypothesized that the ideal device and implantation technique for each individual patient is still lacking, and that a heart team approach in decision-making could tailor the most reasonable therapeutic option, balancing risk and benefit, for each individual patient, in an effort to optimize the outcome. All percutaneous intracardiac occlusion devices have a less invasive implant procedure but require periprocedural OAC/NOACS and a subsequent switch to antiplatelet therapy, ideally for a short period of time. Extracardiac thoracoscopic clipping of the LAA is more invasive, since it demands access to the chest, however, it does not require post-procedural anticoagulant or antiplatelet treatment [7,9].

Percutaneous closure with a Watchman device has been extensively studied in the Protect AF and Prevail trials compared to anti-vit K [11,32]. Only 19% and 29.7%, respectively, of patients at high risk of bleeding (HASBLED > 3) were included. With an implantation rate of 88%, at follow up, 14% of patients at 45 days, 8% at 6 months, and 6.8% at 1 year could not suspend anticoagulation [32], with an incidence of major bleeding of 3.1%, mainly gastrointestinal and intracranial, responsible for 6.2% of all deaths [11]. In the PRAGUE-17 trial, percutaneous appendage closure was compared to NOAC; reporting implantation success of 90%, with 81% of patients on DAPT and 16% on NOACS for 3 months, and subsequent switch to aspirin, with an annual rate of bleeding of 5.5%, device related thrombi of 3.4%, and peri-device leak in 2.2%, requiring a period of anticoagulation [33]. The more recent Ewolution study showed implant success rates of 98.5%, with 4.1% device thrombosis and 4.6% major bleeding. At discharge 16% of patients were still on VKA, 11% on NOAC, 60% on dual APT, and 7% on single APT, while only 6% did not take any antithrombotic therapy [12]. Follow-up at 2 years showed that 8% were still on anticoagulant, 7% on DAPT, and 71% on SAPT [13]. Only 14% were not taking any anticoagulants, which might be a drawback of this treatment option, in case of an underlying condition predisposing to bleeding if on antiplatelet therapy. The recent ASPREE trial showed significantly higher bleeding events in healthy elderly patients taking aspirin [34]. In the ACTIVE trials, the association of clopidogrel and aspirin was less effective than anticoagulation for stroke prevention, by reducing the risk by 28% compared to aspirin alone, but at the expense of increased bleeding [35], adding concerns about this post-procedural pharmacological regime. Off-label endovascular device implantation (without subsequent anticoagulant/antiplatelet therapy) has been reported, but the implications of this decision on safety and efficacy of the procedure must be tested in prospective clinical trials. No large series are available on stand-alone thoracoscopic LAA clipping. A recent publication by our group on 45 patients showed no procedure-related complications, and complete LAA occlusion in all patients with mean stumps of 3.3 ± 2.8 mm. At a mean follow-up of 16.4 ± 9.1 months (range, 2–34), with all patients off NOAC/OAC or APT, no ischemic stroke or hemorrhagic complications occurred [9]. In the largest series, by Ohtsuka et al., on 201 patients (mean age 74 years, mean CHA_2_DS_2_VASc score (±SD) 4.1 ± 1.4, and mean HASBLED score 2.9 ± 1.0) who underwent standalone thoracoscopic stapler and loop LAA removal, all appendages were successfully amputated and no hospital or major procedure-related complications were reported. All patients received one month of anticoagulation, and antiplatelets were prescribed only in those already taking them before surgery. Follow up was completed in 198 patients (98%), and over a mean period of 48 months (range 12–110) two cardiogenic strokes were reported (0.25 event per 100 patient-years) and confirmed by MRI, concluding that their technique was safe and effective for LAA management, providing acceptable mid-term systemic embolism prevention without anticoagulation [36].

In this study, the therapy was chosen according to each patient’s expected outcome and the risk, safety, and efficacy of two standard procedures: standalone totally thoracoscopic LAA exclusion with clipping, or percutaneous closure with a Watchman.

The main variables we found to be essential for selection were the HASBLED score, previous chest surgery, CT scan report, and comorbidities with a known risk of bleeding, documented or not in the medical history (e.g., Rendu–Osler–Weber syndrome, diffuse GI angiodysplasia, cerebral amyloid angiopathy, or cerebral cavernomas). The HASBLED score is a scoring system for bleeding, but over a value of 5, no bleeding risk data are available [18]. A HASBLED score > 3 is considered high risk, corresponding to 3.74 bleeding risk per 100 patient/year, with 8.7 with a value of 4 and 12.5 at 5 [18]. The Ewolution trail showed a trend towards higher hemorrhagic events rates with a HASBLED >3, with bleeding requiring transfusion being the most common complication (13.7%) [12]. Based upon these data, in case of a HASBLED score < 5, with historically known transfusion requirement on APT, or presence of comorbidity with known risk of bleeding if on anticoagulant/antiplatelet therapy (such as in Rendu–Osler–Weber syndrome, diffuse angiodysplasia, or cerebral amyloid degenerative disease), or in general with HASBLED >5, a LAAO-T approach was the first choice. Previous cardiac and lung surgery were relative contraindications for LAAO-T. Potentially difficult single lung ventilation, as in obese patients (BMI > 35), was another variable predisposing to percutaneous management of the LAA. In all other cases a percutaneous procedure was preferred.

Procedural times and average length of hospital stay were almost identical in both groups, as well as the post-procedural ventilation time. Before the procedure, 42% of patients received blood transfusion and of those 76%, were enrolled in LAOO-T, since antiplatelet therapy was deemed to have a negative prognosis in the long term, this may have contributed to there being no hospital readmissions nor blood transfusions during follow up; consequently improving quality of life.

The strict follow up and consultation of the regional healthcare database enabled us to document the absence of neurological/cardiological events and hospital readmissions of any cause, which is in line with previous reports [7,9]. Therefore, the results from this study suggest that endocardial and epicardial LAA exclusion techniques are both valid strategies for a tailored treatment in specific subgroups of patients.

### Limitations

This was a single-center pilot prospective study with a small sample size, and on this basis cannot provide certain conclusions. The length of follow up is a major limitation. The possibility of providing a percutaneous and a totally thoracoscopic program for appendage closure is not available in all cardiac centers for these reasons, the reproducibility of data, the interpretation, and the decision-making processes need to be confirmed in larger studies. Furthermore, the HASBLED score is a validated predictive tool for bleeding, not including disease related bleeding/re-bleeding risk on antiplatelet therapy, and, therefore, the decision-making process of the heart team was based upon variables not selected through statistical analysis and applied to individual clinical data. In conclusion, since a larger randomized controlled study is required to better understand and estimate the risk and benefits, comparing both treatments, the findings of this study should be taken as preliminary.

## 5. Conclusions

A multidisciplinary heart team approach for patients contraindicated for oral anticoagulants and indicated for left atrial appendage closure, offering an epicardial thoracoscopic, as well as an percutaneous endocardial, treatment option, could be a safe and effective strategy for optimizing results and minimizing risks. The main variables we found to be essential for selection were the HASBLED score, CT scan report, lung function previous chest surgery, and comorbidities with known risk of bleeding documented or not in the medical history. These risk profiles and long-term benefits of a LAAO procedure should be taken into account and discussed with the patient when choosing a treatment strategy. Epicardial LAA clipping could be advised in patients in whom the risk of bleeding is estimated as being too high for antiplatelet therapy.

## Figures and Tables

**Table 1:** Patients characteristics.

Clinical Variable	Watchman	Atriclip ProII	*p* Value
Age mean (±SD); median (Q1–Q3)	72.3 (±7.5); 75.5 (65.5–79)	74.9 ± (8); 75 (69.5–79.5)	0.828
Gender (M) (%)	13 (65)	13 (65)	1.000
CHA_2_DS_2_VASc mean(±SD); median (Q1–Q3)	4.2 (±1.5); 4 (3–5)	6 (±1.5); 6.5 (4.5–7)	0.002
HASBLEDmean ± SD; median (Q1–Q3)	3.5 (±1.1); 3 (3–4)	5.4 (±1.4); 5(4–7)	<0.0001
Previous ischemic stroke (%)	3 (15%)	3 (15%)	1.000
Diabetes mellitus (%)	3 (15)	4 (20)	1.000
Hypertension (%)	14 (70)	15 (75)	0.723
Previous cerebral hemorrhage (%)	3 (15)	9 (45)	0.038
GI bleeding	6 (30)	6 (30)	1.000
non cerebral/GI	9 (45)	5 (25)	0.185
Renal disease (CrCl < 30mL/min) (%)	4 (20)	5 (25)	1.000
Dialysis (%)	1 (0.5)	1 (0.5)	1.000
Previous cardiac surgery (%)	4 (20)	0 (0)	0.106
COPD (%)	1 (0.5)	6 (30)	0.092
Carotid stenosis 100% (%)	1 (0.5)	1 (0.5)	1.000
Unilateral carotid stenosis >50% (%)	2 (10)	6 (30)	0.235
Bilateral Carotid stenosis <50% (%)	17 (85)	13 (65)	0.144
Disease with known bleeding risk on aspirin (%)	0 (0)	11 (55)	<0.0001
Type of AF permanent (%)	13 (65)	12 (60)	0.744
Paroxysmal	3 (15)	1 (0.5)	0.605
LS Persistent	4 (20)	7 (35)	0.288
Previous AF ablation	0	0	
EjectionFraction (EF)mean (±SD); median (Q1–Q3)	57 ± 8.7; 58.8 (54.5–60)	53 ± 6.3; 55 (50–56)	0.067
Total	20	20	

**Table 2:** Appendage anatomy.

Type of Appendage	Watchman	Atriclip ProII
Windsock	6	3
Cauliflower	5	6
Cactus	4	5
Chicken Wing	5	6
Total	20	20

**Table 3:** Procedural data.

	Watchman	AtriclipProII	*p*-Value
Operative time mean ± SD; med(IQR)	54.4 ± 12.7; 54 (46.5–57.5)	52.2 ± 14.3; 47.5 (45–55)	0.232
Ventilation time mean ± SD; med(IQR)	11.2 ± 6.4; 11.5 (9.5–13.5)	15.8 ± 16.4; 11 (9.5–13)	0.643
Hospital stay mean ± SD;med(IQR)	3.4 ± 0.7; 3 (3–4)	3.8 ± 0.9; 3.5 (3–4)	0.289
Complication	1groin hematoma	1 pericarditis	
Post-procedure therapy	DAPT for 3 months then SAPT	None	

## Data Availability

The data presented in this study are available on request from the corresponding author. The data are not publicly available due to our hospital patient privacy protection code.
